# Serum Calcium Level Combined with Platelet Count May Be Useful Indicators for Assisted Diagnosis of Extremity Posttraumatic Osteomyelitis: A Comparative Analysis

**DOI:** 10.1155/2021/6196663

**Published:** 2021-10-28

**Authors:** Guan-qiao Liu, Nan Jiang, Yan-jun Hu, Qing-rong Lin, Lei Wang, Bin Yu

**Affiliations:** ^1^Division of Orthopaedics & Traumatology, Department of Orthopaedics, Southern Medical University Nanfang Hospital, Guangzhou 510515, China; ^2^Guangdong Provincial Key Laboratory of Bone and Cartilage Regenerative Medicine, Southern Medical University Nanfang Hospital, Guangzhou 510515, China

## Abstract

**Background:**

A previous study had reported that patients with osteomyelitis (OM) appeared to be more likely to develop hypocalcemia before and after surgery. Calcium sulfate (CS) is frequently used as a local antibiotic vehicle in the treatment of OM, which may also affect serum calcium level. However, whether changes of serum calcium level are caused by OM and/or local use of calcium sulfate remains unclear. Also, platelet (PLT) count plays a crucial predictive role in periprosthetic joint infections (PJIs), but its role in assisted diagnosis of OM is largely unknown. The purpose of this study was to determine whether serum calcium level and PLT count may be helpful in assisted diagnosis of PTOM.

**Methods:**

Between January 2013 and December 2018, we analyzed 468 consecutive patients (392 males and 76 females), including 170 patients with posttraumatic OM (PTOM), 130 patients with aseptic bone nonunion (ABN), and 168 patients recovered from fractures with requirement of implant removal set as controls. Preoperative serological levels of calcium, phosphorus, and PLT were detected, and comparisons were conducted among the above three groups. Additionally, correlations and receiver operating characteristic (ROC) curves were displayed to test whether calcium level and PLT can differentiate patients with ABN and PTOM.

**Results:**

Outcomes showed that the incidences of asymptomatic hypocalcemia (PTOM vs. ABN vs. controls = 22.94% vs. 6.92% vs. 8.82%, *χ*^2^ = 21.098, *P* < 0.001) and thrombocytosis (PTOM vs. ABN vs. controls = 35.3% vs. 13.84% vs. 12.35%, *χ*^2^ = 28.512, *P* < 0.001) were highest in PTOM patients. Besides, the mean serological levels of phosphorus in PTOM and ABN patients were significantly higher than those in the controls (*P* = 0.007). The Area Under the Curve (AUC) of the ROC curve outcomes revealed that, with the combination of serum calcium level with PLT count, the predictive role was acceptable (AUC 0.730, *P* < 0.001, 95% CI 0.681-0.780). Also, serological levels of calcium of 2.225 mmol/L and PLT count of 246.5 × 10^9^/L were identified as the optimal cut-off values to distinguish patients with and without PTOM. However, age- and gender-related differences in serum calcium levels (age, *P* = 0.056; gender, *P* = 0.978) and PLT count (age, *P* = 0.363; gender, *P* = 0.799) were not found to be statistically significant in any groups. In addition, no significant correlations were identified between serum calcium level and PLT count (*R* = 0.010, *P* = 0.839).

**Conclusions:**

Asymptomatic hypocalcemia and thrombocytosis appeared to be more frequent in this cohort with PTOM. Serological levels of calcium and PLT count may be useful biomarkers in screening patients suspected of PTOM.

## 1. Introduction

As one of the most frequent types of extremity chronic osteomyelitis (COM), posttraumatic OM (PTOM) arises as a sequelae or complication following open fractures and orthopaedic surgeries. Currently, this disease still represents great challenges to clinicians because of its long disease course, a higher rate of infection recurrence, and higher risks of physical and psychological disabilities as well [1]. Although many biomarkers have been reported for assisted diagnosis of PTOM [2, 3], such as white blood cell (WBC), erythrocyte sedimentation rate (ESR), C-reactive protein (CRP), procalcitonin (PCT), interleukin-6 (IL-6), and tumor necrosis factor alpha (TNF-*α*), early and accurate diagnosis of such a disorder still imposes great challenges [4].

Calcium (Ca^2+^) plays a crucial role in the intra- and extracellular compartments [5], which is also a key component, along with inorganic phosphate in the skeleton [6]. Therefore, this indicator has been used for assisted diagnosis of many disorders, such as Rickets [7], primary hyperparathyroidism [8], and parathyroid carcinoma [9]. Meanwhile, lower calcium levels have been found in patients with severe COVID-19 infections [10] and neonatal infections [11]. In our previous study, we observed that 16.4% of the PTOM patients suffered from asymptomatic hypocalcemia before surgery [1]. However, potential value and underlying mechanisms of serum calcium level in PTOM diagnosis remain unclear.

Platelets (PLTs) play an important role in the hemostasis and inflammatory response. Previous studies had indicated that a lower PLT count were found in patients with leukemia [12], sepsis [13], and cirrhosis [14], with a relatively higher PLT count among patients with liver cancer [15], iron deficiency [16], rheumatoid arthritis [17], sarcoidosis [18], and inflammatory bowel disease [19]. Besides, patients with NAFLD also tend to have platelet abnormalities as previously described, and platelet indices can be used to aid in the diagnosis of NAFLD as well [20]. Thus, it has been applied for assisted diagnosis of many diseases. Besides, platelet-rich plasma had been used for the treatment of tissue infection [21] and OM [22]. Although it had been reported that high expression of PLT count to mean PLT volume ratio played an important role in the prediction of periprosthetic joint infections (PJIs) [23] and diabetes foot infections (DFIs) [24], potential value of PLT in PTOM diagnosis is still unknown.

The purpose of this study was to investigate potential roles of serum calcium and PLT count in assisted diagnosis of PTOM.

## 2. Patients and Methods

### 2.1. Study Design, Setting, and Data Source

This observational study was performed in a tertiary medical center in Southern China. Patient data were collected from those who were diagnosed with PTOM and aseptic bone nonunion (ABN) from 2013 to 2018 with the age between 18 and 65 years, and those from fracture healing patients were collected from 2017 to 2018 also with the age between 18 and 65 years. Informed consent was waived due to the retrospective design of the present study. However, their personal information was anonymous prior to analysis. The study protocol was approved by the medical ethical committee of the hospital.

### 2.2. Definition, Sample Collection, and Inclusion and Exclusion Criteria

Diagnosis of fracture healing (as the control group) was made clinically and radiologically [25], which contains the following points: (1) there are no tenderness and longitudinal percussion pain in the local part of the fracture, (2) there is no abnormal local activity, and (3) X-ray shows continuous callus at the fractured site and the fracture line has been blurred. Patients were included if they meet the following inclusion criteria: (1) patients met the diagnostic criteria of fracture healing, (2) the patient required removal of the fracture fixation devices, and (3) the patient was admitted to the hospital only because of FH, without comorbidities. Patients were excluded if they meet one of following exclusion criteria: (1) abnormal serological levels of ESR, serum CRP, and WBC, (2) comorbidities that may influence the levels of the biomarkers, and (3) unavailable data or missing data.

ABN was also made clinically and radiologically. ABN is defined [26] as patients with pain and motion at the fracture site, and radiological nonunion as no bone bridging observed for at least 9 months after initial treatment and no visible progressive signs of healing for 3 months. The cases of hypertrophic nonunion included in the study met the criteria of the Weber and Cech classification [27]. Patients are excluded if they had draining fistulas or infection determined by ESR, serum CRP, and WBC levels or if the nonunion was septic [28].

PTOM is defined as a long-term (over 10 weeks [10]) bone infection with or without surrounding soft tissue infection, which occurs as sequelae or a complication following open fractures and/or orthopaedic surgeries. Diagnosis of PTOM is built on any of the following three points: (1) histopathological test of the intraoperative specimens revealing infection; (2) cultures from at least two suspected infection sites showing the same pathogen, and (3) a definite sinus tract/fistula connecting directly the bone or the implant.

Peripheral blood samples were collected from cubital veins of the fasting patients with a tourniquet in the root of the upper arm. The whole procedure of sample collection took approximately 30 seconds. Then, serological levels of calcium, phosphorus, and PLT count were detected. The normal range of these biomarkers' serological levels is as follows: calcium: 2.20-2.65 mmol/L, phosphorus: 0.74-1.39 mmol/L, and PLT count: 100‐300 × 10^9^/L, as provided by the Medical Clinical Laboratory of our hospital.

Eligible patients in the present study were those with definite diagnosis of bone healing, ABN, and PTOM. Additionally, patients included should have normal renal, liver, and parathyroid functions. Excluded from the present study were patients with acute OM, joint arthritis, rheumatoid arthritis, nonextremity bone infection, or hematogenous or DF-associated OM. In addition, patients with abnormal renal function (e.g., glomerulonephritis and renal failure) and liver function (e.g., sepsis and cirrhosis), hyperparathyroidism or hypoparathyroidism, comorbidities (e.g., malignant tumors), iron deficiency, cancer (e.g., lung and breast cancer), tuberculosis, taking antidiabetic and antimalarial medication, consumption of alcohol, hormone deficiency disease (e.g., cortisone deficiency), blood diseases (e.g., leukemia and thrombocytopenic purpura), and taking calcium supplements that may affect serum calcium, phosphorus level, and PLT count were also excluded.

### 2.3. Statistical Analysis

Statistical analysis was conducted by the Statistical Product and Service Solutions (SPSS) 24.0 software (SPSS Inc., Chicago, IL, USA). Data distributions were evaluated for normality using the Kolmogorov-Smirnov test. For normally distributed data, one-way analysis of variance (ANOVA) was used to compare the differences among the three groups. Bonferroni or Dunnett's T3 method was used for post hoc multiple comparisons following the ANOVA test based on the outcomes of the homogeneity test of variance. Otherwise, the Kruskal-Wallis *H* test was used. The chi-square test was used to compare differences of rates among different groups. The optimal cut-off value for the serum calcium level and PLT count that can reliably differentiate patients with and without PTOM was determined using the receiver operating curve (ROC) analysis. The best cut-off point was identified by calculating the Youden index (Youden index = sensitivity + specificity − 1). The PLT count at which the Youden index is maximal is considered the optimal cut-off point. The serum calcium level at which the Youden index is minimal was considered the best cut-off value. Statistically significant difference was defined as *P* value of ≦0.05.

## 3. Results

### 3.1. Patient Demographics

A total of 468 patients (392 males and 76 females) were analyzed, including 170 PTOM patients (147 males and 23 females), 130 ABN patients (101 males and 29 females), and 168 healthy controls (145 males and 23 females). No significant difference was found regarding the gender ratio among the groups (*χ*^2^ = 5.280, *P* = 0.071). In addition, no significant difference was identified regarding the median age among the groups (PTOM group: 40.01 years, IQR 30.50~47.50; ABN group: 38.92 years, IQR 27.50~48.50; and control group: 39.75 years, IQR 30.50~47.00; *P* = 0.716).

### 3.2. Changes of Serum Levels of Calcium and Phosphorus and PLT Count of the Three Groups

Significant differences were identified regarding the mean serum levels of total calcium (*F* = 9.672, *P* < 0.001), phosphorus (*F* = 5.010, *P* = 0.007), and PLT count (*F* = 27.781, *P* < 0.001) among the groups prior to surgery.

**Figure 1:**
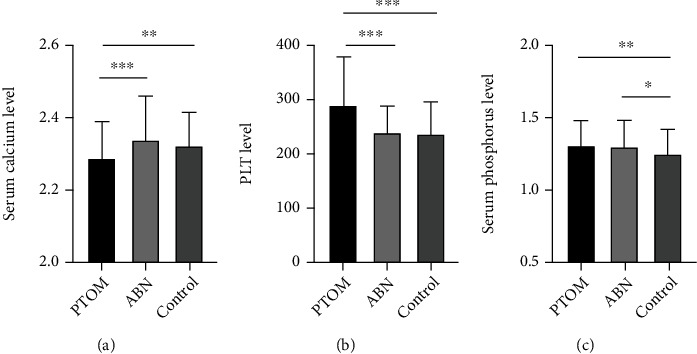
Changes of serum levels of calcium and phosphorus and PLT count in PTOM, ABN, and controlled groups. (a) Representative changes of serum calcium level among the three groups. (b) Representative changes of serum phosphorus level among the three groups. (c) Representative changes of PLT count among the three groups. Data are represented as mean ± SEM. ^∗^*P* < 0.05, ^∗∗^*P* < 0.01, and ^∗∗∗^*P* < 0.001.

As for serum calcium level, outcomes of the post hoc multiple comparisons revealed that, compared with the controlled group of 2.32 mmol/L and the ABN group of 2.34 mmol/L, the mean level of calcium in the PTOM group of 2.28 mmol/L was the lowest (controlled group vs. PTOM group, *P* = 0.002; ABN group vs. PTOM group, *P* < 0.001). However, no statistical difference was identified in the serum calcium level between the ABN group and controlled group (*P* = 0.197) (Figure 1(a)).

Regarding PLT count, outcomes showed that, compared with the controlled group of 234.92 × 10^9^/L and ABN group of 237.64 × 10^9^/L, the mean PLT count of the PTOM group was the highest (287.38 × 10^9^/L) (controlled group vs. PTOM group, *P* < 0.001; ABN group vs. PTOM group, *P* < 0.001). Similarly, there was no significant difference regarding the median PLT count between the ABN group and controlled group (*P* = 0.965) (Figure 1(b)).

With respect to the serum phosphorus level, outcomes revealed that, compared with the controlled group (1.24 mmol/L), the mean phosphorus levels in the PTOM group (1.29 mmol/L) (*P* = 0.005) and ABN (1.29 mmol/L) (*P* = 0.011) were significantly higher, while there was no statistical difference between the ABN group and PTOM group (*P* = 0.906) (Figure 1(c)).

### 3.3. Subgroup Analysis of Serum Calcium Level and PLT Count in the PTOM Group

As age and gender may affect serum calcium level and PLT response [29, 30], subgroup analyses were conducted sorted by age (below and above 40 years) and gender. Because no significant difference was found in the serum level of phosphorus between the ABN group and PTOM group, further stratified analysis was not conducted for this indicator.

**Figure 2:**
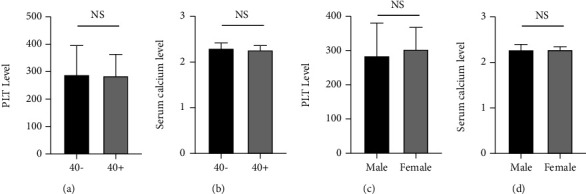
Subgroup analyses of serum calcium and PLT count in the PTOM group. (a, b) Representative changes of serum calcium level (a) and PLT count (b) in different age groups. (c, d) Representative changes of serum calcium level (c) and PLT count (d) in different gender groups. Data are represented as mean ± SEM. NS: not significant. *P* > 0.05.

For age analysis, no significant differences were identified regarding the median serological levels of calcium (*P* = 0.056) or PLT count (*P* = 0.363) between the two age groups (Figures 2(a) and 2(b)). Similarly, no statistical differences were noted regarding serum calcium level (*P* = 0.978) or PLT count (*P* = 0.799) between the males and females (Figures 2(c) and 2(d)). These results indicated that age and gender might have limited influences on serum calcium level and PLT count among PTOM patients.

### 3.4. Correlations and Predictive Values of Serum Calcium Level and PLT Count in PTOM Diagnosis

**Figure 3:**
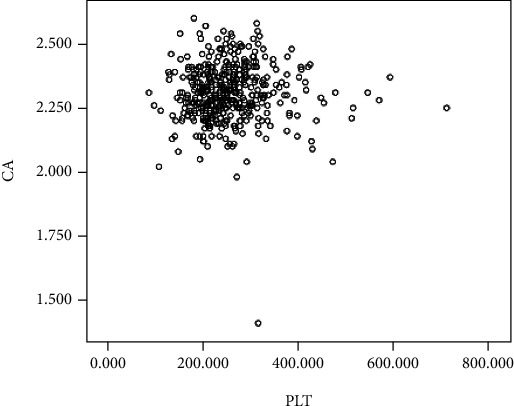
Scatter plot diagram shows the correlation between serum calcium level and PLT count, showing no significant correlations between serum calcium level and PLT count (*R* = 0.010, *P* = 0.839).

Pearson correlation analysis was used to test whether there existed a correlation between PLT count and serum calcium level, and the results showed that no significant correlations were identified between serum calcium level and PLT count among PTOM patients (*R* = 0.010, *P* = 0.839) (Figure 3).

**Figure 4:**
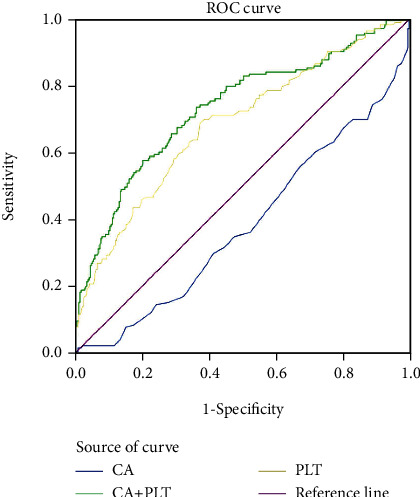
ROC curves of serum calcium level, PLT count, and combined levels. The blue curve shows AUC of the ROC curve of serum calcium level (AUC = 0.618, 95% CI 0.564~0.671). The yellow curve shows AUC of the ROC curve of PLT count (AUC = 0.680, 95% CI 0.628~0.731). The green curve shows AUC of the ROC curve for the combination of serum calcium level and PLT count (AUC = 0.730, 95% CI 0.681~0.780).

As there was no correlation between serum calcium level and PLT count, we analyzed their respective predictive values for PTOM diagnosis.  Figure 4 shows the ROC curve analysis for serum calcium level and PLT count, which was carried out to assess the capacity for discriminating patients with and without PTOM. The AUC of the ROC curve indicated less acceptable accuracy for serum calcium level (blue curve, AUC = 0.618, *P* < 0.001, 95% CI 0.564~0.671) and the PLT count (yellow curve, AUC = 0.680, *P* < 0.001, 95% CI 0.628~0.731). Interestingly, when serum calcium level was combined with PLT count, AUC of the ROC curve indicated acceptable (AUC > 0.7) accuracy (green curve, AUC = 0.730, *P* < 0.001, 95% CI 0.681~0.780). Serum calcium level at 2.225 mmol/L and PLT count at 246.5 × 10^9^/L were identified as the optimal cut-off values.

## 4. Discussion

Serum calcium level and PLT count are common indicators for clinical diagnosis, and recently, some bacterial infectious diseases have been shown to have relationships with decreased serum calcium level and increased PLT count [31–33]. During infection, PLTs interact directly and indirectly with bacteria through a wide range of cellular and molecular mechanisms [32]. Toll-like receptors (TLRs) can recognize different subtypes of lipopolysaccharide (LPS) and respond by releasing cytokines and effective proteins, such as antimicrobial peptides and immunomodulatory molecules, to stimulate another immune cells and clear pathogens [34, 35]. Meanwhile, circulating calcium and its regulators are widely involved in the immune activities [36]. It has been shown that vitamin D (VD), as a regulator of calcium and phosphate levels in circulation, can be converted into calcitriol, the active form of VD, by inflammatory cells and immune cells, which can regulate its differentiation, activation, and proliferation [37]. Moreover, hypocalcemia is more common in patients with Gram-positive and Gram-negative bacterial infections [31], which indicate that calcium may play a crucial role in bacteria-related infection.

In our previous study [1, 38, 39], we found that patients with PTOM were more likely to develop hypocalcemia before and after surgery. However, as calcium sulfate (CS) has been used as a filling material in the treatment of OM, which may affect serum calcium level, we were not clear whether changes of serum calcium level are caused by PTOM or the supplementary CS. In many infectious diseases, it can be accompanied by abnormalities of PLTs and serum calcium level, such as dengue fever [40] and hepatitis A infection [41]. What is more, we also noticed about the predictive role of PLT count in PJIs [23], but there is no related report in PTOM. So, in this study, we collected serum levels of calcium and phosphorus and PLT count from PTOM patients who had sought medical attention in our hospital between 2013 and 2018. In addition, we collected data from patients with ABN and bone healing. We found that, compared with the bone healing group and ABN group, PTOM patients appeared to be more likely to develop hypocalcemia and thrombocytosis. Then, we discussed the predictive value of serum calcium level and PLT count, and we found that using serum calcium level and PLT count separately for predictivity was less effective; however, when combining them together, the predictive value was acceptable. Although the optimal cut-off values of both serum calcium level and PLT count were still within their respective normal ranges, it should be noted that the outcomes were influenced by multiple factors, such as disease terms, previous treatment strategy, and immune status. In addition, the sample size may also affect the outcomes. Nonetheless, our present outcomes demonstrated that the asymptomatic hypocalcemia and thrombocytosis may imply the high risks of PTOM, which provides a new insight into the predictivity of complex and atypical OM in the clinical working.

The current study has several limitations. Firstly, the number of the included participants was still limited. Future studies with a larger sample size are warranted to obtain more accurate outcomes. Secondly, we have not combined the typical inflammation indicators, such as ESR, CRP, and WBC, with serum calcium level and PLT count for OM diagnosis, which may reduce the diagnostic value of serum calcium level and PLT count. Thirdly, we used total calcium as the indicator to diagnose hypocalcemia, and we cannot control the patients eating calcium-rich food or not, where biases may exist. Consequently, cautious attitude should be taken towards the outcomes. Lastly, this study only reported the change of serum calcium level, serum phosphorus level, and PLT count in PTOM patients; whether such changes may occur in another types of COM (e.g., chronic hematogenous and DF OM) remains unclear. Therefore, the predictive value of serum calcium level and PLT count in other types of COM should be further investigated.

## 5. Conclusions

Asymptomatic hypocalcemia and thrombocytosis appeared to be more frequent in this cohort with PTOM. Serum calcium level and PLT count may be helpful indicators for assisted diagnosis of PTOM.

## Data Availability

The datasets of the present study are available from the corresponding authors on reasonable request.
